# The *Arabidopsis thaliana* CLAVATA3/EMBRYO-SURROUNDING REGION 26 (CLE26) peptide is able to alter root architecture of *Solanum lycopersicum *and *Brassica napus*

**DOI:** 10.1080/15592324.2015.1118598

**Published:** 2015-12-15

**Authors:** Nathan Czyzewicz, Ive De Smet

**Affiliations:** a Division of Plant and Crop Sciences, School of Biosciences, University of Nottingham, Loughborough LE12 5RD, United Kingdom; b Department of Plant Systems Biology, VIB, B-9052, Ghent, Belgium; c Department of Plant Biotechnology and Bioinformatics, Ghent University, B-9052, Ghent, Belgium; d Centre for Plant Integrative Biology, University of Nottingham, Loughborough, LE12 5RD, United Kingdom

**Keywords:** CLAVATA3/EMBRYO-SURROUNDING REGION (CLE) peptide; CLE26, oilseed rape, root architecture, tomato

## Abstract

Optimal development of root architecture is vital to the structure and nutrient absorption capabilities of any plant. We recently demonstrated that AtCLE26 regulates *A. thaliana* root architecture development, possibly by altering auxin distribution to the root apical meristem via inhibition of protophloem development. In addition, we showed that AtCLE26 application is able to induce a root architectural change in the monocots *Brachypodium distachyon* and *Triticum aestivum*. Here, we showed that application of the synthetic AtCLE26 peptide similarly affects other important agricultural species, such as *Brassica napus* and *Solanum lycopersicum*.

Optimal development of root architecture is vital to the structure and nutrient absorption capabilities of any plant. Root architecture is inherently plastic to deal with an ever-changing environment.^1,2^ Key parts of root architectural development are primary root growth and post-embryonic development of lateral roots, which increase the available surface area for nutrient absorption.^2,3^ Understanding the role of signaling elements involved in governing root architecture will enable the future adaptation of crop species to maintain and/or increase yield in areas of stress conditions.

One group of signaling molecules involved in a wide array of physiological and biochemical processes leading to development of tissues and organs are post-translationally modified peptides.^4-6^ These relatively short proteins commonly form ligands for receptors and initiate signaling cascades.^5^ Signaling peptides exist as 2 main families, cysteine-rich, and post-translationally modified peptides, with CLAVATA3/EMBRYO-SURROUNDING REGION (CLE) peptides belonging to the latter family.^4^ Post-translationally modified peptides are expressed as precursor sequences, which are then commonly modified by tyrosine sulfation or hydroxyprolination.^4^ Hydroxyprolinated residues can be further modified by addition of l-arabinose, and all of the above modifications are able to modify the activity of the peptide, by altering the secondary structure of the peptide or providing additional surfaces by which interaction between peptide and receptor can occur.^7^

The CLE peptide family has been demonstrated to regulate several aspects of growth and development in *Arabidopsis thaliana*, as well as in several other species, with TRACHEARY ELEMENT DIFFERENTIATION INHIBITORY FACTOR (encoded by CLE41/CLE44), CLE45 and CLE40 being key signals in root architectural development.^5,8-10^ It has recently been demonstrated that AtCLE26 regulates *A. thaliana* root architecture development, possibly by altering auxin distribution to the root apical meristem via inhibition of protophloem development.^9,11^ In addition, it was shown that AtCLE26 application is able to induce a root architectural change in the monocots *Brachypodium distachyon* and *Triticum aestivum*.^11^ Given that enhanced root architecture is key to increasing crop yield, and that the CLE peptide family is present across a broad range of species, we also evaluated if application of the synthetic AtCLE26 peptide was able to similarly affect other important agricultural species.

*Brassica napus* (oilseed rape), is a crop species closely related to *A. thaliana*, and is an important agricultural species, allowing for the cultivation of oils for both human consumption and biofuel generation.^12,13^ Additionally, the meal produced from the seed husks remaining after oil extraction is regularly included in feed for livestock, due to the high protein content (circa 38%).^14^ *B. napus* is a hybrid of 2 diploid progenitor species *B. rapa* and *B. oleracea*.^15^ In order to determine if AtCLE26p was active on this crop species, we germinated and grew seedlings on media containing 1 µM peptide as detailed previously.^11^ This indicated that AtCLE26p is able to inhibit primary root growth and increase lateral root density in *B. napus* (Fig. 1A, C and D), which is phenotypically similar to our previously reported data for AtCLE26 application to *A. thaliana, B. distachyon* and *T. aestivum*.^11^

**Figure 1.**
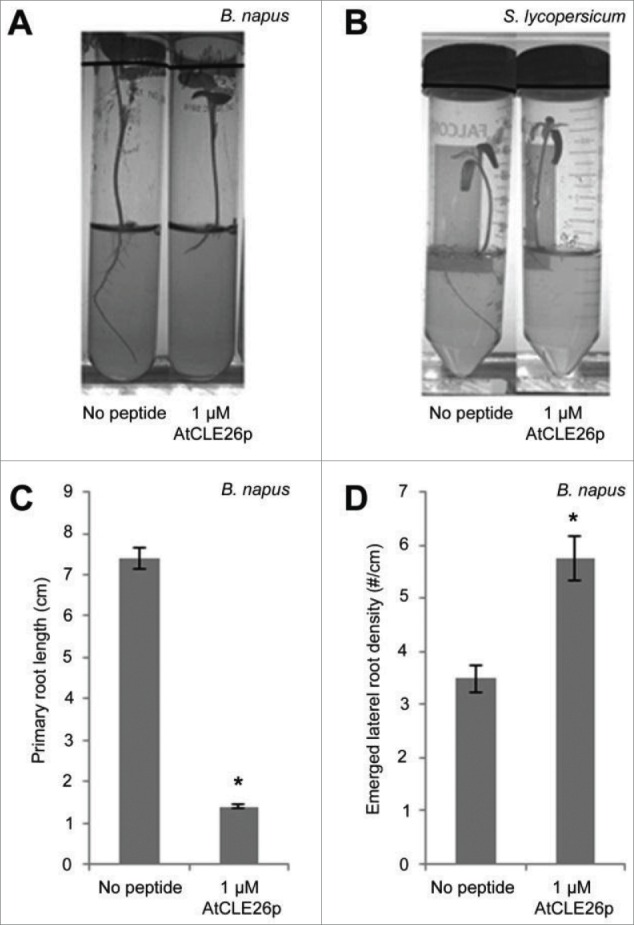
AtCLE26 effect on *B. napus* and *S. lycopersicum* root architecture. (A-B) Representative pictures of *B. napus* (A) and *S. lycopersicum* (B) grown in the absence or presence of 1 µM AtCLE26p. (C-D) Quantification of *B. napus* root phenotypes with respect to primary root length (C) and lateral root density (D) in absence or presence of 1 µM AtCLE26p (n = 18 for no peptide and 26 for 1 µM CLE26). Graphs depict average ± standard error of indicated sample numbers. *, *p*< 0.05 according to Student's t-test.

Since CLE peptide species are present across a broad range of plant species,^11^ we also investigated whether AtCLE26 can affect a larger range of crop species than the closely related *B. napus* and cereal crops. A less closely related, but similarly commercially important crop species is tomato (*Solanum lycopersicum*), which is grown and consumed globally, and is a key species in deciphering the signaling pathways involved in fruit ripening.^16^ In order to determine if AtCLE26p was active on this crop species, we germinated and grew tomato (M82D) seedlings on media containing 1 µM peptide as detailed previously.^11^ Compared with the data reported previously for *A. thaliana, B. distachyon* and *T. aestivum*,^11^ and the results for *B. napus* reported here, growth of *S. lycopersicum* on medium containing AtCLE26p resulted in a similar root phenotype (Fig. 1B).

The morphological change upon application of AtCLE26p to root architecture of *B. napus* and *S. lycopersicum* (Fig. 1) resembles that previously reported for *A. thaliana, B. distachyon*, and *T. aestivum*,^11^ suggesting that *B. napus *and* S. lycopersicum* contain a homologous AtCLE26-activated signaling module that acts to govern root architecture. It is yet to be determined whether the observed morphological change to crop species results from disruption of phloem auxin transport, as observed in *A. thaliana*.^11^ However, given the similarities in root morphology, it is possible that the same process of auxin transport is disrupted in AtCLE26-treated *B. napus*.

Our previous analyses indicated that multiple crop and model species contain putative CLE26 orthologues.^11^ However, since the initial phylogeny did not include *S. lycopersicum*, a BLASTp (Phytozome) of *A. thaliana* mature peptide sequences was conducted on the *S. lycopersicum* proteome, which returned 12 putative tomato CLE peptides. Here, we used the annotated proteome data for *B. rapa* instead of *B. napus*, since there is no annotated proteome data for *B. napus*.^13^ Mature peptide sequences from *A. thaliana, S. lycopersicum* and *B. rapa* were aligned and arranged into a phylogenetic tree using CLC workbench (Fig. 2A–B). Our results indicated that *B. rapa* contained a protein (Bra007891) with high homology to AtCLE26p and with the mature peptide having an identical amino acid sequence to that of AtCLE26p (RKVPRGPDPIHN) (Fig. 2C), which is in agreement with earlier observations.^11^ Since *B. napus* contains the *B. rapa* genome, it is likely that Bra007891 and any potential responsive signaling module represent a homologous CLE26 signaling pathway in *B. napus*. These phylogenetic analyses also revealed that the closest AtCLE26 homolog in *S. lycopersicum* is Solyc05g006610.2.1, with a sequence of RRVPNGPDPIHN, which differs from AtCLE26 at residues 2 and 5, representing a K2R and R5N substitution respectively (Fig. 2C). The Blocks Substitution Matrix (BLOSUM62) indicated that both of these substitutions are likely to be conservative of function (K2R = 2 and R5N = 0).

**Figure 2.**
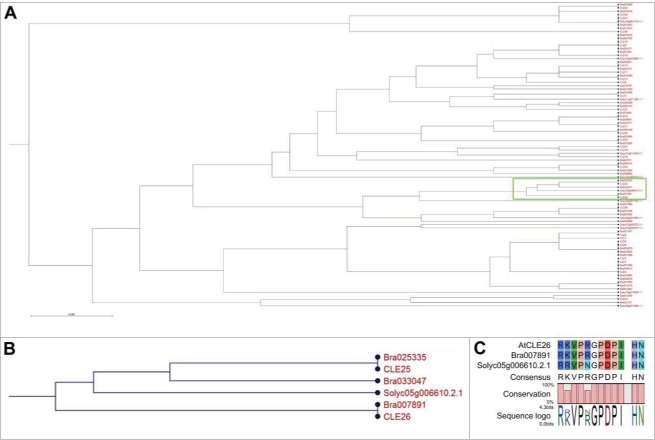
AtCLE26 orthologs in *A. thaliana, B. rapa* and *S. lycopersicum*. (A) Phylogenetic tree of mature CLE peptide sequences. (B) Detail of tree depicted in A, as outlined in green. (C) Sequence alignment of likely AtCLE26 orthologs.

Taken together with our previous findings,^11^ our data indicated that the signaling module initiated by CLE26p (or a functionally related CLE) is conserved across a number of important crop species, suggesting that data generated in the investigation of CLE26 function in *A. thaliana* may potentially impact on a broad range of agriculturally important species. Additionally, further investigation of CLE26 downstream protein effectors may identify putative target proteins for targeted crop improvement, allowing for increased yield of crops in adverse growth conditions.
